# A Comparative Study to Evaluate the Essential Work of Fracture to Measure the Fracture Toughness of Quasi-Brittle Material

**DOI:** 10.3390/ma15134514

**Published:** 2022-06-27

**Authors:** Mohammed Y. Abdellah, Abdul-Rahman Zuwawi, Sufyan A. Azam, Mohamed K. Hassan

**Affiliations:** 1 Mechanical Engineering Department, College of Engineering and Islamic Architecture, Umm Al-Qura University, Makkah 21955, Saudi Arabia; s44280337@st.uqu.edu.sa (A.-R.Z.); saazam@uqu.edu.sa (S.A.A.); mkibrahiem@uqu.edu.sa (M.K.H.); 2 Mechanical Engineering Department, Faculty of Engineering, South Valley University, Qena 83523, Egypt; 3 Production Engineering and Design Department, Faculty of Engineering, Minia University, Minia 61111, Egypt

**Keywords:** fracture, linear elastic, laminates, mode I, quasi-brittles

## Abstract

In the present work, three different woven composite laminates were fabricated using the hand lay-up method. The woven reinforcement fibres were carbon fibres (CFRP), glass fibres (GFRP-W) and (GFRP-R) in combination with epoxy resin. Then, the central notch specimen tensile test (CNT) was used to measure the fracture toughness and the corresponding surface release energy ($G_{IC}$). Then, the data were compared with the essential work of fracture (we) values based on the stored energy of the body to obtain a new standard fracture toughness test for composite laminates using relatively simple techniques. In addition to an extended finite element model, XFEM was implemented over a central notch specimen geometry to obtain a satisfactory validation of the essential work of fracture concepts. Therefore, the average values of ($G_{IC}$) were measured with CNT specimens 25.15 kJ/m^2^, 32.5 kJ/m^2^ and 20.22 kJ/m^2^ for CFRP, GFRP-W and GFRP-R, respectively. The data are very close as the percentage error for the surface release energy measured by the two methods was 0.83, 4.6 and 5.16 for carbon, glass and random fibre composite laminates, respectively. The data for the fracture toughness of XFEM are also very close. The percentage error is 4.6, 5.25 and 2.95 for carbon, glass and random fibre composite laminates, respectively. Therefore, the fundamental work of the fracture concept is highly recommended as a fracture toughness test for composite laminates or quasi-brittle Material.

## 1. Introduction

Quasi-brittle material is characterised by an intermediate zone before the crack tip between fully linear material (brittle) and non-linear material (ductile) [1,2,3]. The fracture toughness of quasi-brittle materials, such as tough ceramics, ice and reinforced laminates, is of great importance for the description and characterisation of their applications [4]. These types of materials are particularly sought after in applications where specific gravity is an issue, such as aerospace [5], marine, offshore [6] and automotive [7]. Fracture toughness or crack resistance is one of the most important properties as it has a great influence on the damage and failure mechanisms of such composite structures. Linear elastic fracture mechanics has introduced many standard specimens with different shapes and geometries for fracture toughness testing, such as compact tension, centre notch tension, edge notch tension and single edge notch bending [8]. There is no standard for reinforced composite laminates. The compact tensile test has been commonly used for fracture toughness testing of unidirectional fibre composite laminates as reported in [9,10], while the central notch tensile specimen (CNT) test was introduced for multidirectional composite laminates by Soutis et al. [11] due to its simplicity and applicability.

The essential work of fracture (EWF) method uses a different criterion that does not depend on the LEFM and J-integral concepts, but on the strip length and the total work and energy stored in a cracked specimen [12]. In [13], the total work carried out by the action of the load in the fracture processing zone was divided into two parts: the essential work (we) corresponding to elastic deformation and the non-essential work *(*wp*)* corresponding to plastic deformation. For the LEFM concept, the EWF *(*we*)* is indicative of the crack propagation resistance or fracture toughness ($G_{IC}$) [14], but it is different from the value of the J-integral responsible for the concepts of elastic plastic fracture mechanics EPFM [15]. It should be recognised that the EWF *(*we*)* takes into account the crack initiation resistance ($G_{IC}$), if the variation in ($G_{IC}$) is small [14], then the relationship between the EWF *(*we*)* and the J-integral ($J_{i}$) is valid according to the EPFM [16]. On the other hand, EWF was characterised by its simplicity in data reduction, sample preparation and even evaluation, which made it attractive for measuring the fracture toughness of ductile thin films [17,18,19]. 

EWF was used early to measure the fracture toughness of thin plastic films, thin coatings and paints [20]. In addition, it was recommended by Martinez et al. [21] as a useful method for thin polymer materials. Recently, the EWF method was used by Pegoretti et al. [22] to measure the fracture toughness of low-density polyethylene films, reporting that the prepared notch affected the results of the linear regression coefficient of EWF and the non-essential work of fracture was lower than that of EWF. Moreover, the increase in specimen thickness led to a propagation of the constriction zone in front of the crack tip, so that the fracture toughness ($J_{C}$), the EWF and the crack opening motion were influenced by the thickness [23,24]. The ligament length was not chosen arbitrarily. When the fracture toughness was tested using the EWF method on low-density polyethylene, Zn and Al alloys, it was concluded that the exact choice of ligament length affected the generation of the linear regression [25]. In addition, other works [18,26,27] measured the fracture toughness with EWF to standardise it for polymeric materials with thin or relatively thick thickness. On the other hand, the loading rate influenced the EWF evaluation for polymeric materials as cited by CHING, Emma CY et al. [28]. Furthermore, the EWF approach was extended to polymeric materials reinforced with carbon nanotubes, as in Tehran et al. [29], where the nanomaterial had an effect on reducing the fracture toughness measured by EWF assessment. Similarly, an opposite effect on the essential work of fracture and non-essential work of fracture parameters was found when nanofillers were added to the low thickness polymer composite material [30,31]. In an experiment by Hassan et al. [32], the concept of EWF was used to model the fracture toughness of hybrid composite laminates used in micromechanical systems. This type of material, where a thin copper layer was attached to a substrate of glass fibre composite laminates, behaves with a large plastic zone, making EWF an acceptable method for sandwich composite structure.

A numerical model proposed by Abdellah [33] used two finite element models, one called the extended finite element method based on a meshless free and an enhancement function, and another based on the J-integral method to correlate the relationship between the mode I fracture toughness ($J_{IC}$) and the EWF of ductile thin aluminium plates. Cohesive zone models, where a fictitious crack was introduced through the model, were used early in combination with FEMs, such as the cohesive element [34,35,36,37] and cohesive surface [36,38,39]. They were characterised by their robustness and good accuracy, but required more computational time. In addition, recently there was a novel and strong mesh-free technique that can be used for stress/rupture analysis (e.g., measurement of fracture toughness) of anisotropic media. This is the element-free Galerkin method for three-dimensional propagation based on a phase field model, which was proposed by Y. Shao et al. [40]. There was also a new technique, the Bezier-based multistep method, which was first derived for a 1D stress intensity factor problem based on the solution of the fourth-order differential equations of LEFM. This model was extended to 2D problems by Kabir, H., and Aghdam, M. M. [41].

As explained earlier, EWF is commonly used to measure the fracture toughness of very ductile, thin polymers with a fully failing softening zone. Therefore, it was extended to involve the composite-reinforced laminates. 

The main idea of the present study is to obtain a simple standard method for measuring the fracture toughness of composite laminates with a mean plastic zone in front of the crack tips. To evaluate this main idea, the following objectives should be established: (1) measure the fracture toughness of carbon, glass and random fibre composite laminates using conventional standard specimens with a central notch, (2) develop an essential work of fracture (EWF) evaluation procedure to obtain alternative fracture toughness values, (3) creation of an extended finite element model (XFEM) to predict the fracture toughness of the reinforced/epoxy composite laminates using a standard tensile specimen with a central notch and (4) final comparison of the two methods to determine the applicability of the EWF aspects for measuring the fracture toughness of quasi-brittle materials such as composite laminates.

The methodology of the article is structured as follows: In the first section, the fundamentals of EWF are described and outlined. In the second section, the experimental matrix is presented and the fabrication techniques are explained. Furthermore, in the third section, the XFEM was derived and implemented using a linear elastic model for centre notch tension specimen. In the fourth section, the results of the fracture toughness tests are presented, discussed and compared. Finally, a conclusion is given with suggestions for the future study.

## 2. Analytical Model (Essential Work of Fracture)

**Figure 1:**
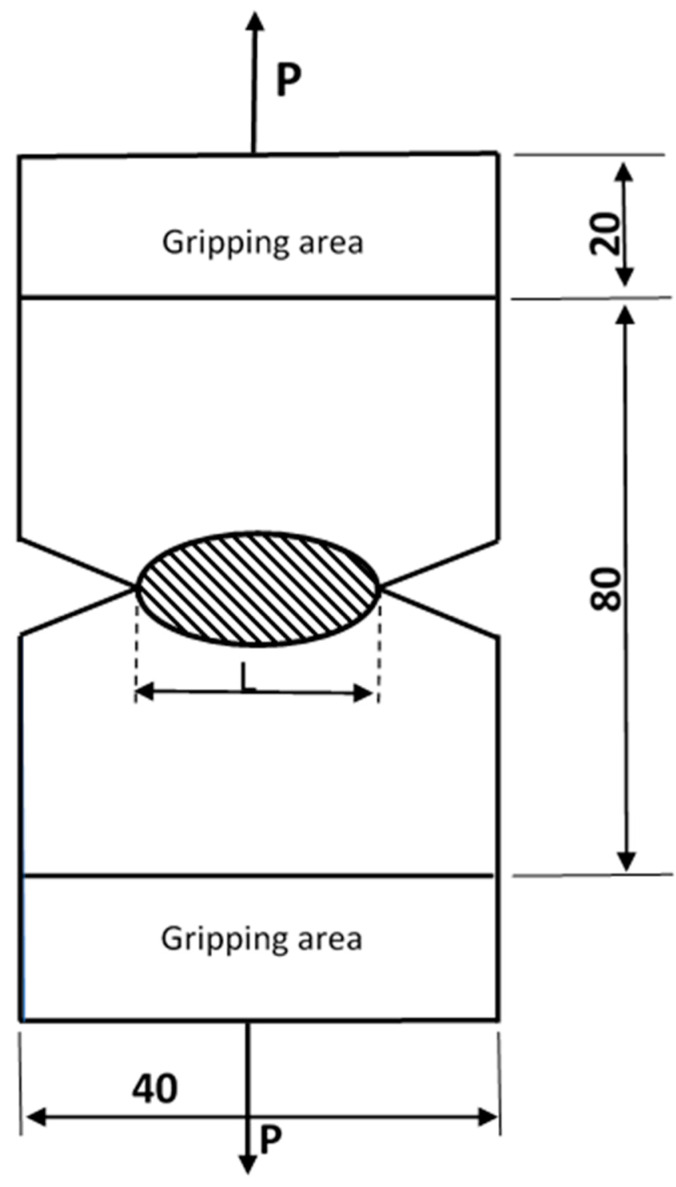
Double edge notch tension (DENT).

**Figure 2:**
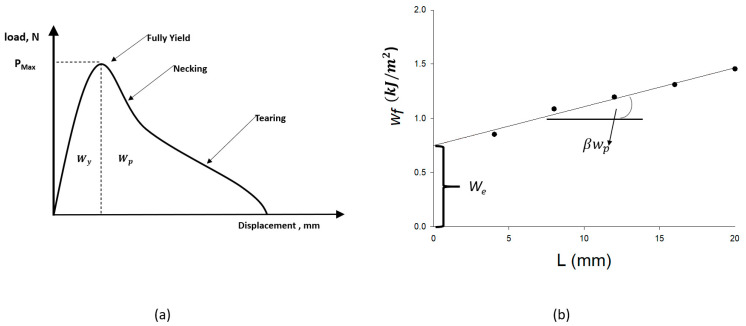
Essential work of fracture (**a**) load–displacement curve and (**b**) EWF fitting.

The EWF method should be defined to understand the current model derivation that links EWF *(*we*)* to the critical J-integral ($J_{IC}$). New concepts are used to describe new assumptions. The EWF system is based on the work of Mai and Cotterell [42] and the recommendation of Broberg [43]. They proposed to divide the total energy consumed in ductile cracking into the work related to the formation of a developed fracture surface (referred to as “essential” work) and the work related to plastic deformation (referred to as “non-essential” work, which depends on the geometry of the plastic deformation). Figure 1 illustrates the procedure for determining the work required by creating a fracture surface in ordinary DENT specimens. The specimen is loaded in tension until the strip *L* yields completely at the maximum load and the two plastic zones that form at the crack tips come into contact [44]. Ductile cracks progress through the ligament until complete failure occurs. Figure 2a shows the curve of load versus displacement δ. As shown in Equations (1) and (2) [13,43,45,46], the total work of fracture ($W_{f}$) is defined as the sum of ($W_{e}$), the essential term, and ($W_{p}$), the non-essential term:

(1) $$W_{f}=\int_{0}^{\delta }p\;d\delta$$

(2) $$W_{f}=W_{e}+W_{p}$$

where ($W_{e}$) refers to the crack tip’s instability and offers surface release work in the crack process zone and ($W_{p}$) refers to the plastic deformation zone behind the fracture process zone. In addition, δ is the failure displacement.

For a given specimen thickness, the surface work of liberation ($W_{e}$) is proportional to the length of the ligament *L*. ($w_{p}$) denotes the volume energy proportional to the volume ($L^{2}t$). To obtain an equation for the energy, divide Equation (2) by the ligament area (*Lt*) as follows:

(3) $$w_{f}=\frac{W_{f}}{Lt}=w_{e}+\beta w_{p}L$$

where β is the shape factor of the plastic deformation and ($w_{p}$) is the specific non-essential work of the fracture and is considered as the plastic work per unit volume of the plastic deformation zone before the crack tip. In addition, we are the release energy of the surface required to initiate the formation of the fracture surface. The relationship of ($w_{f}$) given in Equation (3) is a linear regression related to the ligament length *L*. The positive intercept at *L* = 0 is the specific EWF, ($w_{e}$). The slope of the regression line is determined by fitting the data linearly with the non-essential work of fracture ($w_{p}$) (see Figure 2b).

For a DENT specimen, Equation (2) can be rewritten as follows after the application of a load and complete yielding of the strip:

(4) $$W_{f}=W_{y}+W_{pp}$$

where ($W_{y}$) is the elastic zone’s mechanical energy and ($W_{pp}$) is the plastic zone’s plastic energy consumed for necking and subsequent tearing, as shown in Figure 2a.

($w_{e}$) may divide the elastic zone of EWF; ($w_{y}$) associated with crack initiation and the plastic zone of EWF; and ($w_{pp}$) associated with ripping before necking ahead of the crack tip using Equation (4) as follows:

(5) $$w_{e}=w_{y}+w_{pp}$$

In addition, the slope can be divided as follows:

(6) $$\beta w_{p}=\beta yw_{py}+\beta pw_{pp}$$

where βy and βp are the geometric shape factors related to the plastic zone during ligament yielding and tearing after necking, respectively.

## 3. Methods and Material

### 3.1. Material and Manufacturing

**Figure 3:**
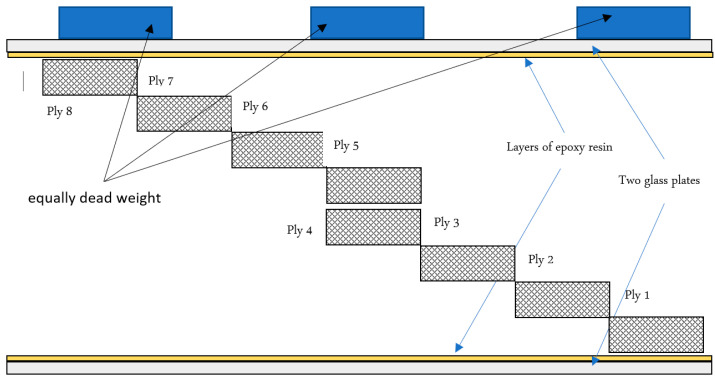
Schematic drawing of laminate setup.

Three different types of woven fibres are used as reinforcing material: carbon fibres, glass fibres and random fibres, while the matrix material is epoxy resin. The properties of these components are listed in Table 1 [47,48]. The composite laminate was constructed using the hand lay-up technique, which is the cheapest and most economical method [49,50]. The hand lay-up technique can be summarised as follows: (1) Two glass plates are used, with one plate serving as a base and coated with wax as a release agent to prevent sticking. (2) First a layer of epoxy is applied, followed by a layer of woven fibres, again a layer of epoxy is spread evenly with a brush and an aluminium roller is used to remove voids. (3) Repeat the previous step with the clean fibre layers until eight layers and the laminate are built up (Figure 3). (4) The second glass plate with release agent is placed over the entire layer. (5) Finally, a series of weights is placed over the second plate to obtain a relatively uniform thickness. The glass plate was removed after 24 h and the laminates were fully cured at room temperature for 21 days [51]. This system of curing offers ease of processing, high reliability and does not require thermal energy [52]. The mixing ratio was as recommended by the manufacturers, i.e., resin: hardener = 2:1 by weight. The fibre volume fraction was measured using the ignition technique according to the ASTM D3171-99 standard [53]. The mean values of volume fraction were found to be 65% for carbon-fibre-reinforced polymer (CFRP), 45% for glass-fibre-reinforced polymer (GFRP) and random-glass-fibre-reinforced polymer (RGFRP). The symbols S1, S2 and S3 refer to CFRP, GFRP and RGFRP, respectively. The mean thickness (t) was 2.5 mm, 5 mm and 4 mm for S1, S2 and S3 materials, respectively.

### 3.2. Un-Notch Tensile Test

**Figure 4:**
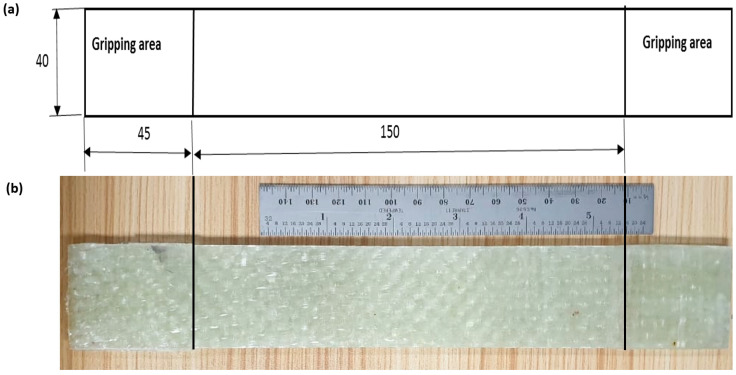
Tensile test specimen geometry; (**a**) Schematic drawing (dimensions in mm); (**b**) Real image.

The un-notch tensile test was performed according to ASTM D3039 [57]. The test is performed on CFRP, GFRP and RGFRP to determine the mechanical properties. The tests were carried out with H-series hydraulic materials testing machines (Zwick/Roell type: Z600H) [58] with a maximum load capacity of 600 kN at a control speed of 2 mm/min. The schematic representation of the specimen geometry can be seen in Figure 4a. The aluminium taps are placed at both ends of the specimens in the clamping area to prevent slippage and damage to the specimens under the action of the clamping force. Real photos of glass-fibre-reinforced epoxy laminates are shown in Figure 4b.

### 3.3. Centre Notch Test

There are many standards for fracture toughness testing based on concepts of linear elastic fracture mechanics to determine the crack tip. The simplest standard based on the model developed by Soutis and Flick [11] is the central notch specimen tensile test. It has been recommended for multidirectional composite laminates. The procedures consist of measuring the peak load at crack propagation of a certain value of the precrack, then the remote failure stress $\sigma_{p}$ is measured. Then, the fracture toughness $K_{IC}$ is calculated using Equation (7) as follows [50]:

(7) $$K_{IC}=\sigma_{p}\times \sqrt{\pi a}\;\sqrt{sec\left(\frac{\pi a}{w}\right)}$$

In addition to the corresponding surface release energy ($G_{IC}$) can be calculated by Equation (8) as follows:

(8) $$G_{IC}=\frac{K_{IC}}{E_{eq}}$$

**Figure 5:**
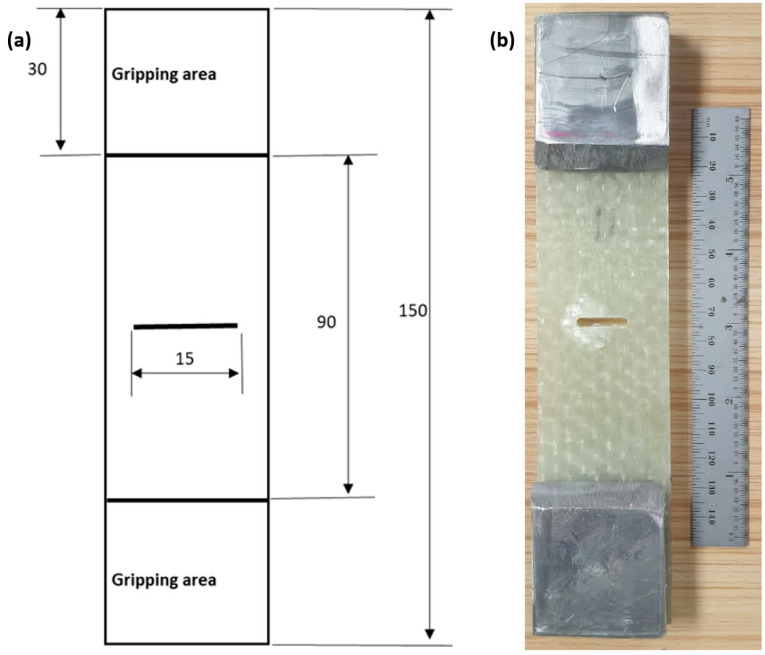
Centre notch tension specimen. (**a**) Schematic drawing (dim. in mm); (**b**) Real image.

The notch must be machined with a cutter or diamond saw and a crack must be made in the centre of the specimen by tapping or sawing with a fine razor blade. See the sample geometry and dimensions in Figure 5. Five samples with a width of 45 mm and a central crack of 15 mm were used for the test matrix. The specimen geometry and dimensions (see Figure 5a) can be produced with a quartz disc saw. Five specimens with a width of 45 mm and a central crack of 15 mm with an approximation width of 3 mm, as recommended by Soutis and Flick [11], were used for the test matrix. The measurement height was chosen to be 90 mm according to J.C. Newman & and M. Jordan Haines [59] (see Figure 5b). Aluminium taps were placed in the clamping area to prevent damage to the machine jaws. The tensile test was carried out with a computerised universal testing machine (Zwick/Roell type: Z600H) [58] with a maximum load capacity of 600 kN at a control speed of 2 mm/min, with both load and displacement recorded by computer during the test.

### 3.4. Extended Finite Element Method XFEM

The XFEM was proposed by Belytschko and Blak [60] based on the work of Melenk and Babuska [61]. It is characterised by neglecting the need to modify the mesh as the crack progresses [62]. Therefore, the analysis of fracture toughness and stress intensity factor can be performed in a shorter time while the crack propagates with considerable accuracy [63]. Therefore, it enables crack modelling independently from the mesh. In XFEM, the finite element unit and the enhancement function (Equation (10)) are divided as follows [62]:

(9) $$u^{h}=\underset{i\in I}{\sum }u_{i}N_{i}\left(x\right)+\underset{i\in I}{\sum }a_{i}N_{i}H\left(x\right)+\underset{i\in k1}{\sum }N_{i}\left(x\right)\left(\overset{4}{\underset{l=1}{\sum }}b_{i,1}^{l}F_{1}^{l}\left(x\right)\right)\;+\underset{i\in k2}{\sum }N_{i}\left(x\right)\left(\overset{4}{\underset{l=1}{\sum }}b_{i,1}^{l}F_{2}^{l}\left(x\right)\right)$$

where *l* is the node in the mesh, $u_{i}$ is degree of freedom, $N_{i}$ is shape function related to node *i*, $a_{i}$ is node crack length, (i∈I) is the subset of nodes enriched by the Heaviside function H(x), (i∈k1) and (i∈k2) are the set of nodes to enrich to model crack tips numbered 1 and 2, respectively, and $b_{i,1}^{l}$, $b_{i,2}^{l}$ are degrees of freedom of node 1 and 2. While the asymptotic crack-tip functions $F_{2}^{l}\left(x\right)$ can be calculated using Equation (11) as follows:

(10) $$F_{2}^{l}\left(x\right)=\left\{\sqrt{r}\sin\left(\frac{\theta }{2}\right),\sqrt{r}\cos\left(\frac{\theta }{2}\right),\;\sqrt{r}\sin\left(\frac{\theta }{2}\right)\sin\left(\theta \right),\;\sqrt{r}\cos\left(\frac{\theta }{2}\right)\sin\theta \;\right\}$$

Each function in these equations has its role in predicting the fracture toughness and stress intensity factor. The work is carried out over the mean notch of the specimen to simulate the fracture toughness or the corresponding surface release energy ($G_{IC}$), but according to LEFM the simulation measures the J-integral around the crack tip from the problem of singularities of the radius $\sqrt{r}$.

#### XFEM Extraction

**Figure 6:**
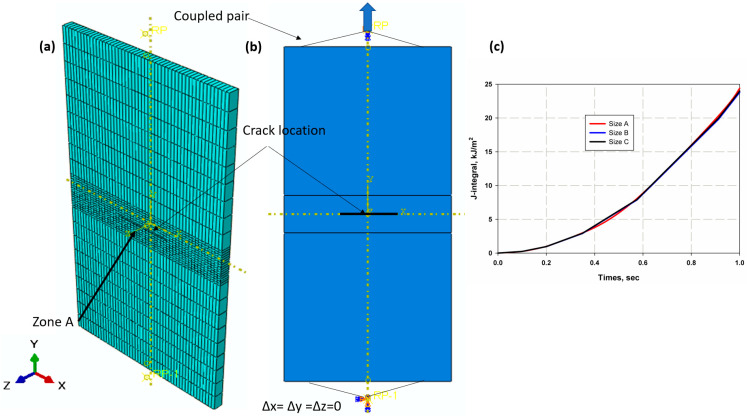
XFEM domain (**a**) mesh, (**b**) boundary condition and (**c**) mesh convergence.

The model was created with ABAQUS 6.11, a commercial software. The finite element domain is shown in Figure 6a, while the mesh and load domains are shown in Figure 6b. It is a rectangular plate with dimensions 90 mm × 45 × thickness, which are 2.5 mm for S1, 5 mm for S2 and 4 mm for S3 (see Figure 6a). In XFEM, a stationary crack was used to measure five J-integrals around the crack tops. The crack was created and inserted in the central zone A as a straight, plane strand of 15 mm length with the same specimen thickness. The initial geometry of the specimens and the initial shape of the precrack or crack length had no effect on the fracture toughness for stationary cracks as reported in [11,64,65], which reduces the number of specimens needed (only one crack length of the specimen is used). This form is preferable in FEM as the strain field around the crack tip becomes singular and the singularity improves the accuracy of the analyses. In addition, 1650 C3D8R elements with a global size of 4.5 were used. The shape of the hexagonal elements was chosen using the swept meshing technique to create a more accurate and dense mesh. The mesh in area A was finer than that of the other plate areas with a global size of 0.9. Three mesh sizes were investigated to obtain an optimal mesh size: 930 (A), 990 (B) and 1650 (C) elements. The initial boundary conditions were a displacement control that completely restricted the movement in all directions at the lower end, while allowing it to move in the y-direction at the upper end. The longitudinal load (F) was applied through the upper ends (see Figure 6c). The load was chosen according to the maximum failure load suggested by the experimental results of CNT. It was 16.5 kN, 29 kN and 10.2 kN for S1, S2 and S3 materials, respectively. The damage criterion was the maximum principal stress (Maxps) theory. Therefore, the maximum principal stress of an un-notch tensile strength was 303 MPa, 187.5 MPa and 125 MPa for materials S1, S2 and S3, respectively. The maximum traction displacement was chosen as the criterion for damage development. At this stage, it was the maximum crack opening $\delta_{cr}$ at which the maximum stress was evident. Therefore, the model extracted by Hahn and Rosenfiled [66] and Perez [67] was selected to calculate the critical crack opening $\delta_{cr}$ using Equation (12) as [68] follows:

(11) $$\delta_{cr}=t\times \varepsilon_{f}$$

The woven fabric composite laminates are commonly simulated using isotropic elastic with longitudinal young modulus of 27.13 GPa, 15.36 GPa and 5.01 GPa for S1, S2 and S3 materials, respectively, whereas a passion ratio equally considers all materials and was selected as 0.34. The materials used in the XFEM are listed in Table 2, these materials were obtained from the simple tension test. In addition, the XFE models can be surmised Additionally, in Table 2, the core subroutine of the code was illustrated in the Appendix A.

### 3.5. Essential Work of Fracture Test

**Figure 7:**
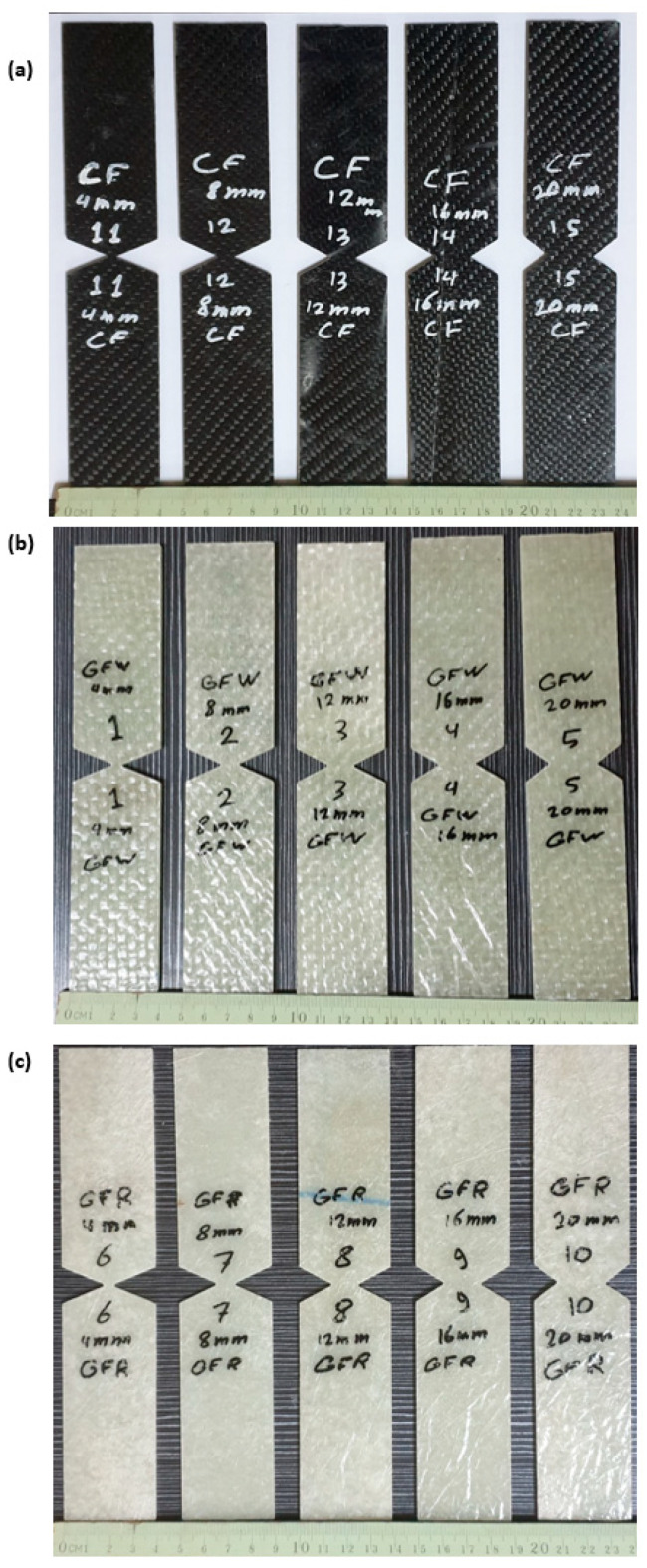
Ideal image of EWF specimens (**a**) CFRP (s1), (**b**) GFRP-w (s2) and (**c**) GFRP-W(s3).

The essential work of the fracture testing methodology was carried out using a DENT specimen with an average width of 40 mm, as described in [12,70] (see Figure 7). The specimen DENT was selected according to the recommendations of [42,71,72,73]. The chosen shape helps to prevent buckling due to symmetry, as the buckling effect is not acceptable as it leads to a large decrease in the tensile load. The tensile load is applied to both ends of the specimens until complete failure. The tensile load is applied at a controlled crosshead speed of 2 mm/min according to [44]. The central crack is created with a 1 mm-thick steel saw disc. The material has a width of 40 mm and a thickness of 2 mm for the S1 and S3 specimens and 5 mm for the S2 specimens. Five specimens were used for EWF values according to the recommendations of [74] for different strip lengths of 4, 8, 12, 16 and 20 mm at room temperature. The mechanical work W under the load–displacement curve would be calculated as follows:

(12) $$W_{f}=\int_{0}^{\delta }p\;d\delta$$

where δ are displacements at failure, respectively, and p is the applied load. The obtained total energy $W_{f}$ (measured using Equation (4)) was plotted against ligament length *L*.

## 4. Results and Discussion

**Figure 8:**
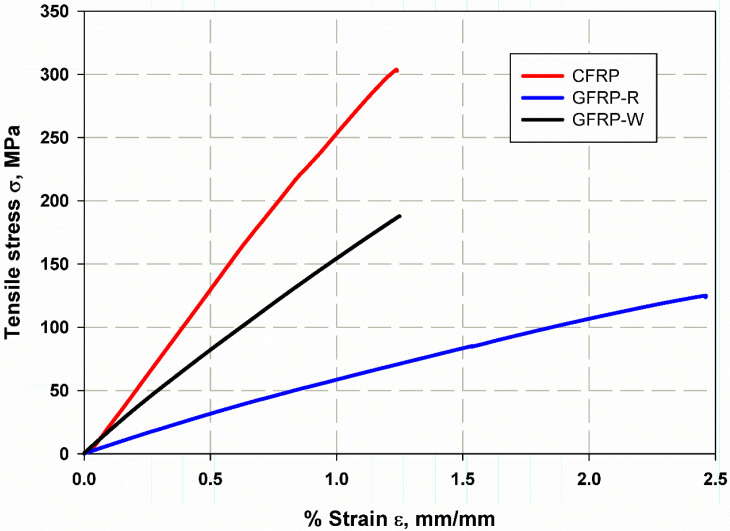
Stress and strain relation for un-notch tensile strength of composite laminates.

**Figure 9:**
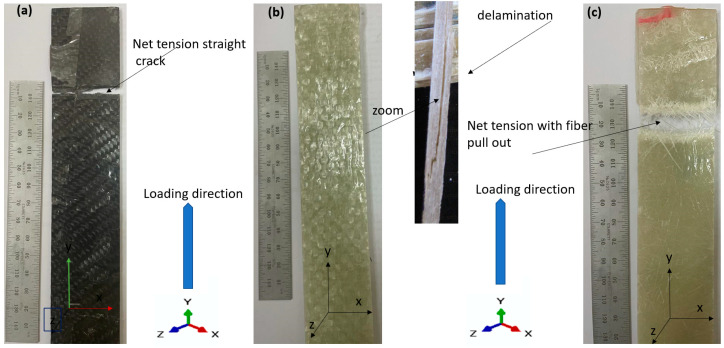
Modes of failure (**a**) CFRP, (**b**) GFRP-W and (**c**) GFRP-R.

Figure 6c shows the relationship between the J-integral and time. It can be seen that for an elastic XFEM model for a stationary crack, the mesh refinement has less influence. The convergence of the mesh has been studied in many papers [4,33,63] and is not significantly affected in the simulation with XFEM, which is a great advantage. Therefore, it was not necessary to investigate the accuracy of the convergence of the mesh in a complex way. The stress–strain relationship of the composite laminates is shown in Figure 8. It was found that S1 of CFRP has an increasing average strength of 303 MPa with a standard deviation SDV 17.98 MPa and a modulus of elasticity of 27.13 GPa with SDV 1.7 GPa, corresponding to (GFRP-W) a lower average tensile strength of 187.5 MPa with SDV 10.5 MPa for GFRP S2 and a modulus of elasticity of 15.36 GPa with SDV of 1.19 GPa, despite the lower values in the case of GFRP-R S3, where the average tensile strength decreased to 125 MPa with SDV of 2.5 MPa and the corresponding modulus of elasticity was very low at 5.01 GPa with SDV of 1.5 GPa. This is due to the fact that carbon fibres have higher stiffness and strength than woven glass fibres or even random fibres. The percent elongation of material S3 with random fibre (GFRP-R) is almost 2.43 larger than that of material S1 with carbon fibre (CFRP), which is due to the fact that carbon fibre has higher stiffness and lower ductility, while material S3 with woven glass fibre has intermediate percent elongation values. The failure modes show net stress for both S1 and S2 (see Figure 9a,c), while for the S1 material with carbon fibres, a straight crack path is observed with fibre breakage at the crack surfaces, while for the S3 material with disordered fibre direction, the net stress is associated with fibre pull-out because the fibres have many directions and possibly due to the lower adhesion between the disordered glass fibres compared to carbon fibres. In addition, the S2 material with woven glass fibres has no failure zone (see Figure 9b) because the failure occurs in the thickness as delamination, which is due to the relative increase of 5 mm in thickness compared to other materials.

### 4.1. Centre Notch

**Figure 10:**
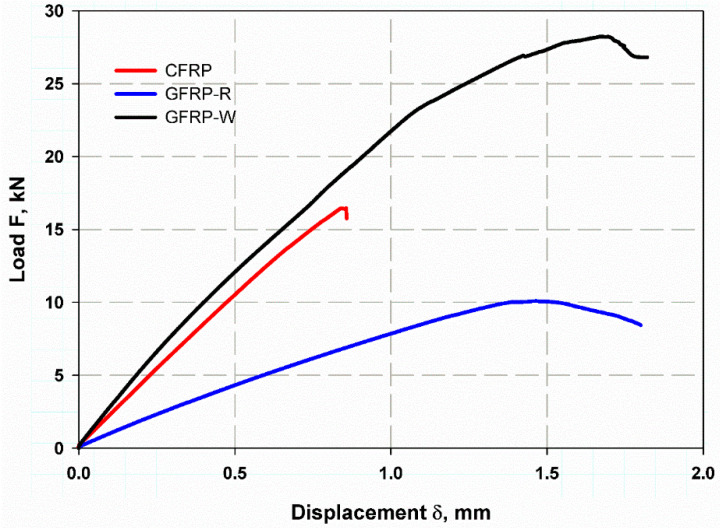
Load and displacement curve of CNT.

**Figure 11:**
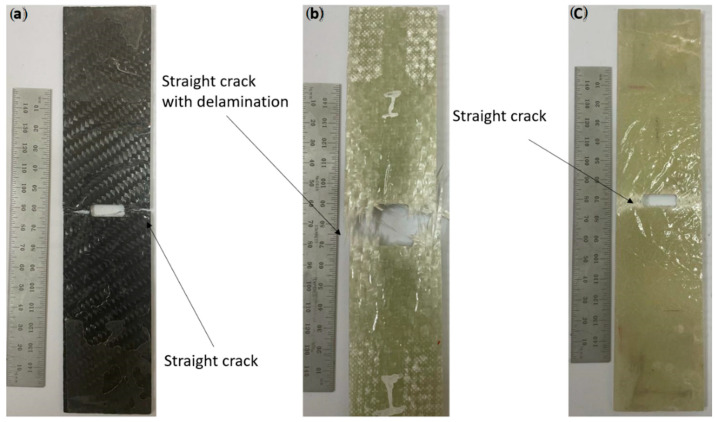
Modes of failure in CNT (**a**) CFRP, (**b**) GFRP-W and (**c**) GFRP-R.

The relationship between load and displacement was shown in Figure 10 for the tensile test on a specimen with a medium notch. The CFRP specimens show a brittle behaviour with a very small softening range, while this was relatively larger for GFRP-R or GFRP-W. This is due to the greater thickness of GFRP-R and GFRP-W compared to the thickness of CFRP, which increases the cross-sectional area. The 5% odorous load PQ would be more suitable for calculating the maximum crack propagation stress $\sigma_{p}$. The average values were 146.5 MPa with SDV 6.17 MPa, 128.6 with SDV 5.6 MPa and 56.11 MPa with SDV 4.4 MPa for CFRP, GFRP-W and GFRP-R, respectively. By substituting these stresses into Equations (7) and (8), you can measure both the fracture toughness $K_{IC}$ and the surface energy ($G_{IC}$). The average values of ($G_{IC}$) were therefore 25.15 $\mathrm{kJ}/m^{2}$, 32.5 $\mathrm{kJ}/m^{2}$ and 20.22 $\mathrm{kJ}/m^{2}$ for GFRP-W and GFRP-R, respectively. The failure modes are shown in Figure 11. All specimens were net stress (see Figure 11a,c), but due to the greater thickness of GFRP-W, the failure mode delamination was observed (see Figure 11b).

### 4.2. Essential Work of Fracture Approach

**Figure 12:**
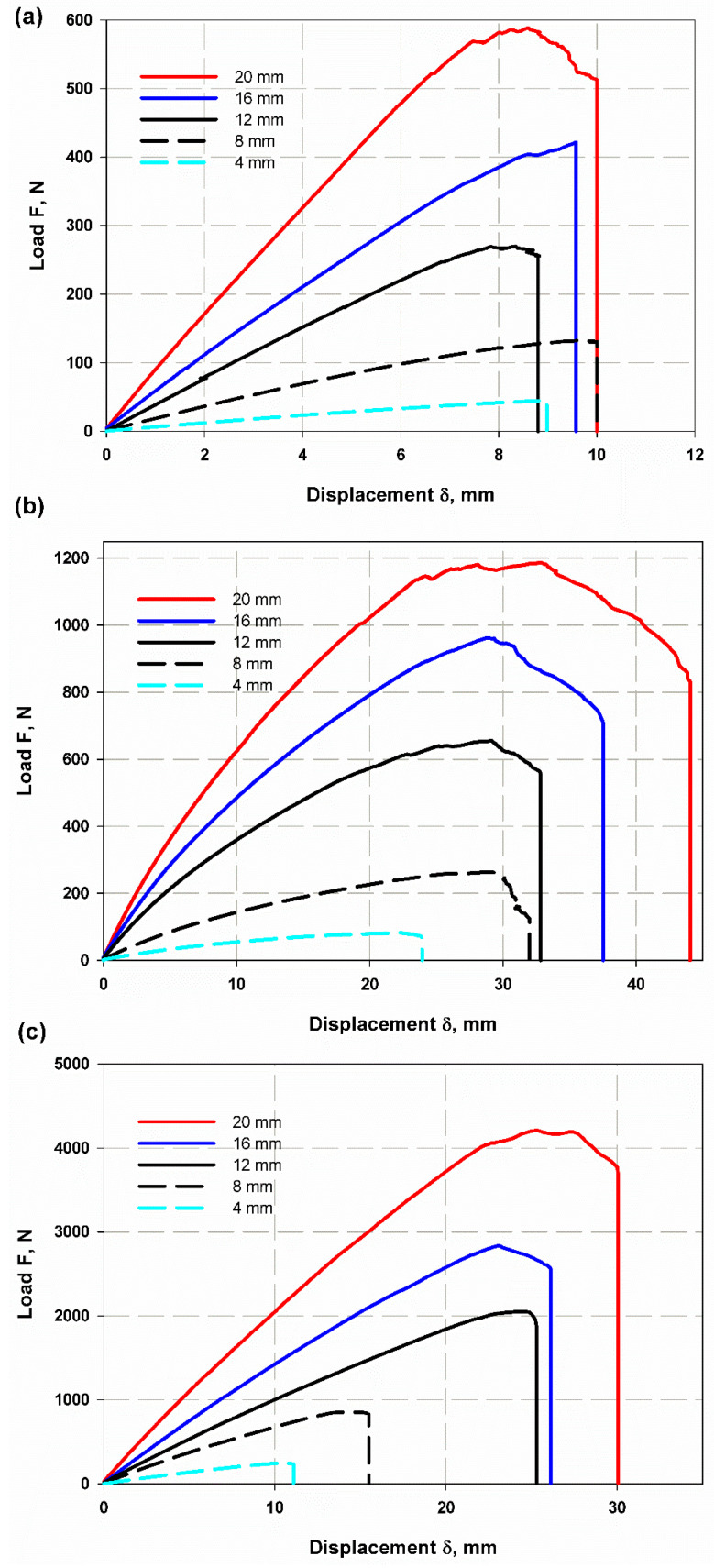
Load and displacement curve in EWF (**a**) CFRP, (**b**) GFRP-W and (**c**) GFRP-R.

**Figure 13:**
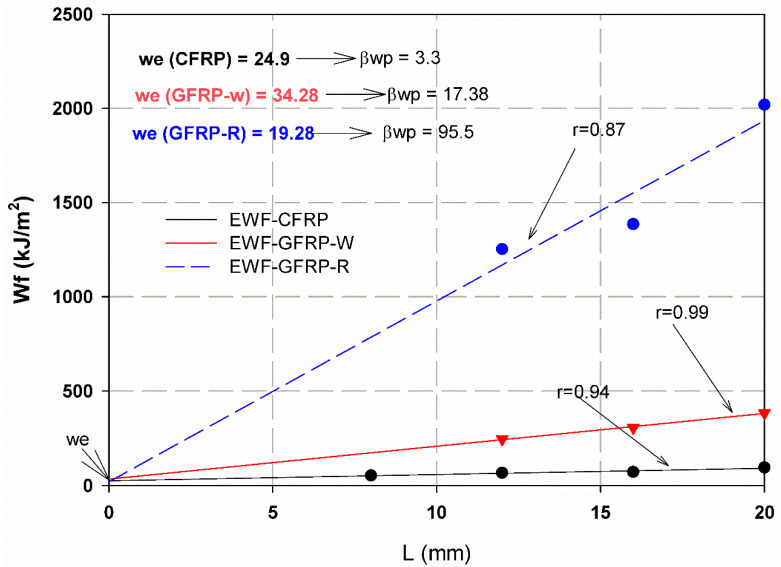
Energy and ligament relation for EWF approach.

**Figure 14:**
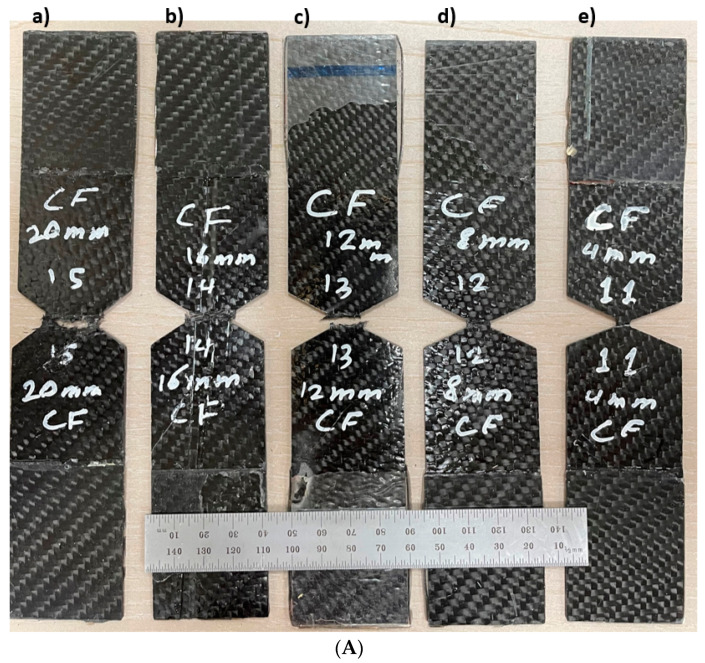

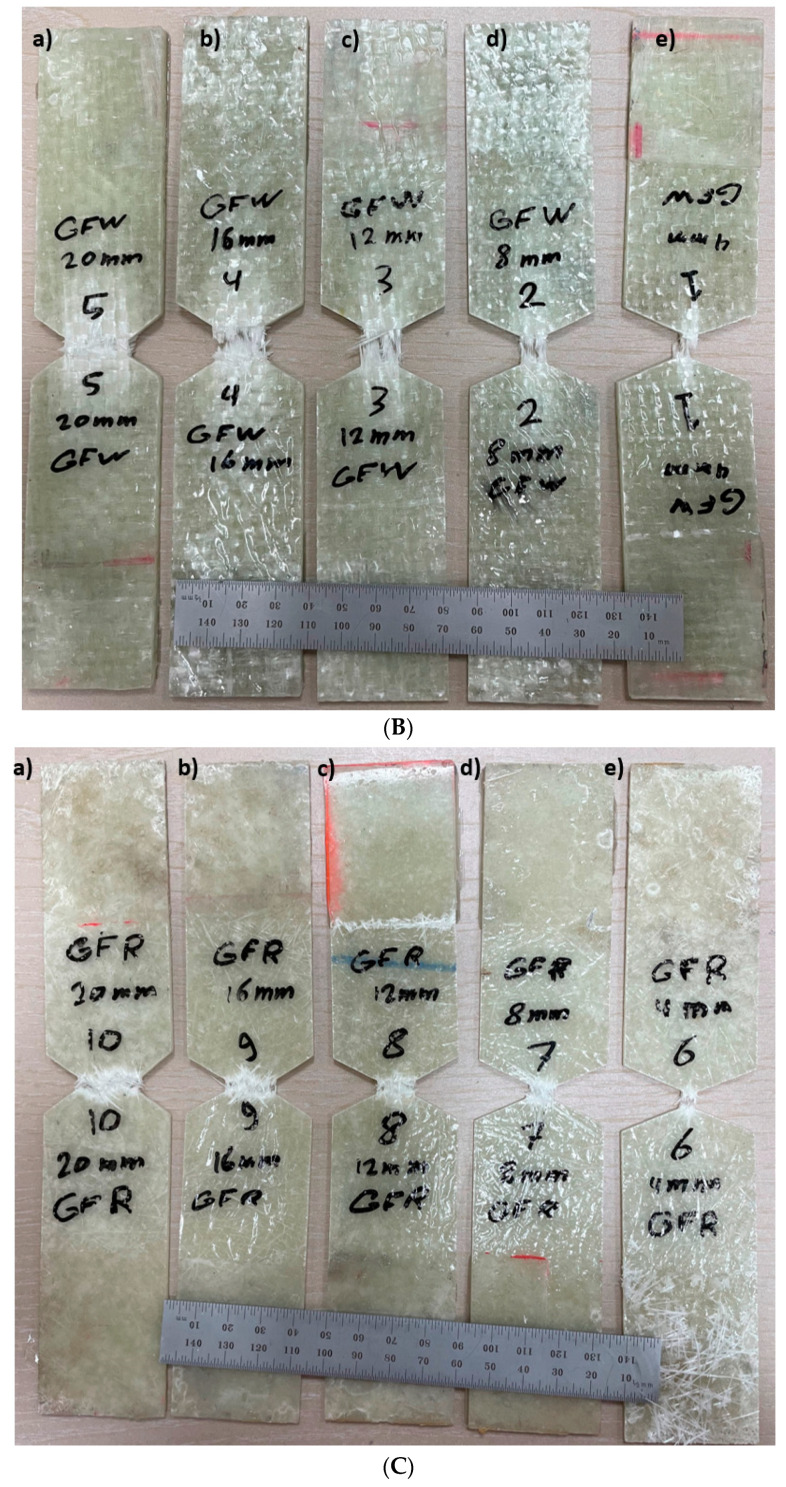
(**A**) Failure modes in EWF test of CFRP: (**a**) 20 mm, (**b**) 16 mm, (**c**) 12 mm, (**d**) 8 mm and (**e**) 4 mm. (**B**) Failure modes in EWF test of GFRP-W: (**a**) 20 mm, (**b**) 16 mm, (**c**) 12 mm, (**d**) 8 mm and (**e**) 4 mm. (**C**) Failure modes in EWF test of GFRP-R: (**a**) 20 mm, (**b**) 16 mm, (**c**) 12 mm, (**d**) 8 mm and (**e**) 4 mm.

The relationship between load and displacement for the essential fracture work EWF is shown in Figure 12a–c for one sample each of CFRP, GFRP-W and GFRP-R. The total work carried out ($W_{f}$) is measured (Equation (9)) as the area under the curves and then divided by the ligament area (Lt) to obtain the total energy per area ($W_{f}$) using Equation (3). Therefore, the essential work of fracture *(*we*)* was presented in the linear regression of Figure 13. The essential work *(*we*)* was 24.93 $\mathrm{kJ}/m^{2}$ with an SDV of 3.6 $\mathrm{kJ}/m^{2}$, 34.28 $\mathrm{kJ}/m^{2}$ with an SDV of 3.4 $\mathrm{kJ}/m^{2}$ and 19.28 $\mathrm{kJ}/m^{2}$ with an SDV of 3 $\mathrm{kJ}/m^{2}$ for CFRP, GFRP-W and GFRP-R, respectively. As reported by [42], to ensure linear regression, the ligament length should not be less than 3–5 times the plate thickness. Therefore, the ligament lengths used to calculate the EWF vary according to the thickness of the samples. If we return to Figure 13, we see that the softening zone, which is responsible for the part of the non-essential fracture work, is relatively small. As mentioned earlier, for quasi-brittle materials such as composites, the plastic zone before the crack tip lies between brittle material (linear material) and ductile material (non-linear material). The slope of each line in Figure 14 illustrates the plastic (softening) part or the non-essential work. It has higher values of 95.5 for GFRP-R than for other materials and a lower value of 3.3 for CFRP, while the intermediate value is 17.38 for GFRP-W. The failure modes are shown in Figure 14A–C. Net stress with straight crack surface without delamination affected CFRP and GFRP-R (see Figure 14A,C), but delamination was observed through the thickness of GFRP-W specimens (see Figure 14B), which is due to their relatively larger thickness. In addition, fibres were pulled out from the GFRP-W and GFRP-R specimens, while fibre breakage was observed in CFRP, which can be attributed to the relatively high stiffness and good debonding ability between carbon fibres and epoxy resin. Table 3 listed all maximum forces and corresponding stresses for all tested specimens and it was shown that the maximum stress values were for specimens S3.

### 4.3. Comparison between Methods

**Figure 15:**
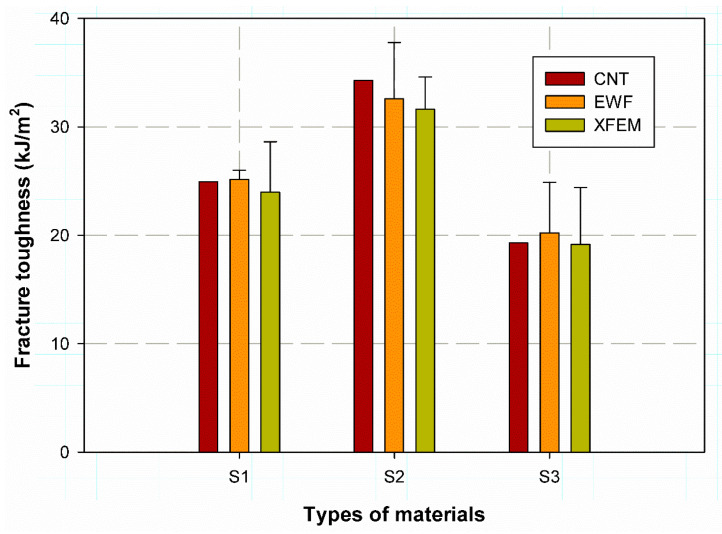
Comparison of EWF with other methods.

The EWF data agree well with the data of the standard ASTM fracture toughness test specimens. To obtain satisfactory results, the XFEM results for the CNT specimens were also used (see Table 4). Figure 15 shows the comparison between the three models. It was also found that the XFEM is robust and good at predicting the fracture toughness of the different woven fibre laminates, with a lower percentage error of 4.65%, 2.97% and 5.25 % for CFRP, GFRP-W and GFRP-R, respectively. Moreover, it was clearly observed that the percentage error was very close when comparing the EWF with the experimental results of the CNT samples, namely 0.83%, 5.16% and 4.64% for CFRP, GFRP-W and GFRP-R, respectively. The average percentage EWF error for CFRP was the lowest at 0.83, which can be attributed to the fact that the lower thickness and high stiffness reduces the softening zone and also reduces the overall effect per area (see Figure 12a). The lowest percentage error of 2.97 was for GFRP-W when XFEM was implemented, as XFEM was based on the initial load introduced in the model subroutine. It was also found that the EWF method is applicable for measuring the fracture toughness of composite laminates. This method is characterised by its simplicity and does not depend on LEFM, where the problem of singularities, crack tips and other geometry factors can affect the accuracy of the results. In addition, the CNT specimens and other standard specimen geometries based on LEFM, such as compact stresses, require special equipment and relatively expensive preparation sequences and procedures. Furthermore, the difference between the fracture toughness of S1 and S2 was found to be almost 28% and between S1 and S3 almost 20%. The S2 material has higher values for fracture toughness than S2 and S3, which is due to a higher amount of epoxy resin than the other samples, although the damage mode delamination by these types of material is not preferred.

## 5. Conclusions

Fabric-reinforced epoxy laminates have more competitive properties as a quasi-brittle material than other multidirectional composite laminates with the same fibre types. This is due to their relatively symmetrical properties. The fracture toughness of the three fabric laminates CFRP, GFRP-W and GFRP-R was measured to be 25.14 kJ/m^2^, 32.59 kJ/m^2^ and 20.22 kJ/m^2^, respectively. The EWF assessment was found to be applicable to these types of brittle material with a small plasticity zone in front of the crack tips. The average accuracy of the method was 3.54% compared to a standard CNT specimen shape and 4.5% compared to the numerical concepts of the XFEM method. In conclusion, it was reported that EWF evaluation can be considered a standardised technique for woven quasi-brittle material. This study can be extended to metal matrix composites and sandwich laminates; moreover, simulation with FEM can include the main fracture concepts to predict the numerical values of EWF. The EWF can also be compared to other standard geometries used in measuring fracture toughness to provide a valuable table for a variety of geometries and materials. Despite these advantages, there are limitations to the use of this method, such as the relationship between strip length and thickness, the accuracy of the fitting regression and the effect of sample size on fracture toughness.

## Figures and Tables

**Table 1:** Mechanical and physical properties of E-glass fibre and epoxy resin [54,55,56].

Properties	E-Glass	AS4-Carbon Fibre	Kemapoxy (150RGL)
Density (kg/m^3^)	2600	1790	1.2
Tensile strength (MPa)	3450	4270	85
Tensile modulus (GPa)	80	228	2.5
Passion ratio	0.25	0.34	0.35
In plane shear modulus (GPa)	30.8	25	1.24

**Table 2:** All XFE model parameters and materials.

Material	Young Modulus (GPa)	Tensile Strength, (MPa)	Passion Ratio [69]	Applied Load (B.C) kN	Element Type	Number of Elements	Evolution Dis. $\delta_{cr}$
CFRP (S1)	27.13	303	0.34	16.5	C3D8R	1650	0.03
GFRP-w (S2)	15.36	187.5	0.34	29	C3D8R	1650	0.125
GFRP-R (S3)	501	125	0.34	10.2	C3D8R	1650	0.03

**Table 3:** Comparing the double edge notch specimens with each ligament length.

Specimens	Load (kN)					Stress (MPa)				
Ligament length (mm)	4	8	12	16	20	4	8	12	16	20
CFRP (s1)	39	134	267	418	585	0.48	1.66	3.34	5.225	7.313
GFRP-W (s2)	82	257	644	962	1171	0.41	1.29	3.22	4.81	5.85
GFRP-R (s3)	244	853	2028	2835	4205	3.26	3.05	10.66	25.35	35.44

**Table 4:** Comparison between fracture toughness measured using CNT, XFEM and EWF.

Material Type	Fracture Toughness, $G_{IC},\;\mathrm{kJ}/m^{2}\;(\mathrm{CNT})$	Fracture Toughness, $G_{IC},\;\mathrm{kJ}/m^{2}\;(\mathrm{XFEM})$	% Error	Fracture Toughness, $we,\;\mathrm{kJ}/m^{2}\;(\mathrm{EWF})$	% Error
CFRP (s1)	25.14	23.97	4.65	24.936	0.83
GFRP-W (s2)	32.59	31.62	2.97	34.28	5.16
GFRP-R (s3)	20.22	19.16	5.25	19.28	4.64

## Data Availability

The data presented in this study are available on request from the corresponding author.

## Nomenclature

δ	Displacement at failure
βy	Geometric shape factors related to the plastic zone during ligament yielding
βp	Geometric shape factors related to the plastic zone during tearing after necking
$\beta w_{p}$	Slope of linear fitting regression
β	Plasticity shape factor
t	Thickness of the specimen
p	Applied load
dδ	Displacement increment
L	Ligament length
a0	Pre-crack length
$\sigma_{u}$	Un-notched tensile strength
$\delta_{o}$	0.2% offset displacement
$w_{y}$	Essential work of the fracture in the elastic zone
$w_{p}$	Non-essential work of the fracture
$w_{e}$	Essential work of the fracture
$W_{y}$	Elastic energy of the elastic and yielding ligament length
$W_{py}$	Relative energy in plastic and yielding of ligament length
$W_{pp}$	Relative plastic energy in tearing and necking
$W_{p}$	Energy of the tearing and necking of the plastic zone
$W_{f}$	Total strain energy attributed to the fracture
$J_{i}$	Initiation J-integral in EPFM
$J_{CFE}$	Finite element J-integral
$J_{C}$	Critical J-integral or release energy in elastic plastic fracture mechanics (EPFM)
$G_{o}$	Initiation surface release energy in LEFM or crack initiation resistance
$G_{IC}$	Surface release energy or critical mode I fracture toughness

## Appendix A

The XFEM input files

**PARTS *Preprint, echo=NO, model=NO, history=NO, contact=NO **PARTS *Part, name = Part-1 *Element, type=C3D8R * *Nset, nset=Part-1-RefPt_, internal *Nset, nset=Set-1 *Surface, type=ELEMENT, name=Surf-1 *Surface, type=ELEMENT, name=Surf-2 ** Section: Section-1 *Solid Section, elset=_PickedSet15, material=Material-1 *End Part *Part, name=crack *End Part ** ASSEMBLY *Assembly, name=Assembly *Instance, name = Part-1-1, part= Part-1 *End Instance *Instance, name = Part-2-1, part= Part-2 *End Instance ** Constraint: Constraint-1 *Coupling, constraint name=Constraint-1, ref node=_PickedSet12, surface= Part-1 *Kinematic -1.Surf-1 ** Constraint: Constraint-2 *Coupling, constraint name=Constraint-2, ref node=_PickedSet16, surface= Part-1-1.Surf-2 *Kinematic *Enrichment, name=Crack-1, type=STATIONARY CRACK, elset=_PickedSet19 *End Assembly ** MATERIALS *Material, name=Material-1 *Damage Initiation, criterion=MAXPS 303., *Damage Evolution, type=DISPLACEMENT 0.01, *Density 1.5e-09, *Elastic 27130., 0.3 Part-1-1.443, 3,Crack-1, -0.335333 Part-1-1.443, 4,Crack-1, -0.335333 Part-1-1.443, 5,Crack-1, 0.335333 Part-1-1.443, 6,Crack-1, 0.335333 Part-1-1.443, 7,Crack-1, -0.335333 Part-1-1.443, 8,Crack-1, -0.335333 Part-1-1.458, 1,Crack-1, 0.335333 Part-1-1.458, 2,Crack-1, 0.335333 Part-1-1.458, 3,Crack-1, -0.335333 Part-1-1.458, 4,Crack-1, -0.335333 Part-1-1.458, 5,Crack-1, 0.335333 Part-1-1.458, 6,Crack-1, 0.335333 Part-1-1.458, 7,Crack-1, -0.335333 Part-1-1.458, 8,Crack-1, -0.335333 Part-1-1.473, 1,Crack-1, 0.335333 Part-1-1.473, 2,Crack-1, 0.335333 Part-1-1.473, 3,Crack-1, -0.335333 Part-1-1.473, 4,Crack-1, -0.335333 Part-1-1.473, 5,Crack-1, 0.335333 Part-1-1.473, 6,Crack-1, 0.335333 Part-1-1.473, 7,Crack-1, -0.335333 Part-1-1.473, 8,Crack-1, -0.335333 Part-1-1.488, 1,Crack-1, 0.335333 Part-1-1.488, 2,Crack-1, 0.335333 Part-1-1.488, 3,Crack-1, -0.335333 Part-1-1.488, 4,Crack-1, -0.335333 ** BOUNDARY CONDITIONS ** Name: BC-1 Type: Symmetry/Antisymmetry/Encastre *Boundary _PickedSet22, ENCASTRE ** ---------------------------------------------------------------- ** STEP: Step-1 *Step, name=Step-1, inc=100000 *Static 1., 1., 1e-10, 1. ** BOUNDARY CONDITIONS ** Name: BC-2 Type: Displacement/Rotation *Boundary _PickedSet23, 1, 1 _PickedSet23, 3, 3 _PickedSet23, 5, 5 _PickedSet23, 6, 6 ** LOADS ** ** Name: Load-1 Type: Concentrated force *Cload _PickedSet20, 2, 16500. ** CONTROLS *Controls, reset *Controls, parameters=time incrementation , , 20, 20, 20, 20, 20, 20, , , ** OUTPUT REQUESTS *Restart, write, frequency=0 ** FIELD OUTPUT: F-Output-1 *Output, field *Node Output CF, PHILSM, PSILSM, RF, U *Element Output, directions=YES LE, PE, PEEQ, PEMAG, S, STATUSXFEM *Contact Output CDISP, CSTRESS *Output, history, frequency=0 ** HISTORY OUTPUT: H-Output-1 *Contour Integral, crack name=Crack-1, contours=5, xfem *End Step
